# Effect of oxygen vacancy and highly dispersed MnO_*x*_ on soot combustion in cerium manganese catalyst

**DOI:** 10.1038/s41598-023-30465-7

**Published:** 2023-02-28

**Authors:** Yi Zhu, Zhen Chen, Hongmei Li, Quan Wang, Xingyu Liu, You Hu, Cuimei Su, Rui Duan, Shanhu Chen, Li Lan

**Affiliations:** 1 College of Chemistry Biology and Environment, Yuxi Normal University, Yuxi, 653100 China; 2 Institute of Biology and Environmental Engineering, Yuxi Normal University, Yuxi, 653100 China; 3 College of Chemistry and Chemical Engineering, Jiangxi Science and Technology Normal University, Nanchang, 330013 China; 4 College of Materials and Mechatronics, Jiangxi Science and Technology Normal University, Nanchang, 330013 China

**Keywords:** Chemistry, Catalysis, Heterogeneous catalysis

## Abstract

Cerium manganese bimetallic catalysts have become the focus of current research because of their excellent catalytic performance for soot combustion. Two series of cerium manganese catalysts (Na-free catalysts and Na-containing catalysts) were prepared by coprecipitation method and characterized using XRD, N_2_ adsorption–desorption, SEM, Raman, XPS, H_2_-TPR, O_2_-TPD, Soot-TPR-MS and in situ IR. The effects of abundant oxygen vacancies and surface highly dispersed MnO_*x*_ on soot catalytic combustion of cerium manganese catalysts prepared by different precipitants were analyzed. The activity test results show that the active oxygen species released by a large number of oxygen vacancies in the cerium manganese catalyst are more favorable to the soot catalytic combustion than MnO_*x*_ which is highly dispersed on the surface of the catalyst and has good redox performance at low temperature. Because the catalytic effect of MnO_*x*_ on the surface of Na-free catalysts is more dependent on the contact condition between the catalyst and the soot, this phenomenon can be observed more easily under the loose contact condition than under the tight contact condition. The activity cycle test results show that these two series of catalysts show good stability and repeated use will hardly cause any deactivation of the catalysts.

## Introduction

Soot particles emitted by diesel engines can not only cause air pollution and haze, but also easily invade human respiratory system due to their small size, moreover, the heavy metals and organic matter absorbed by them can cause serious diseases^1–3^. Diesel particulate filter (DPF) with a filtration efficiency of up to 90% is an effective means to control soot emissions^4^. The initial temperature of soot combustion is higher than 450 °C, and the burnout temperature is higher than 650 °C, so it is not conducive to spontaneous combustion of soot within the exhaust temperature range of diesel engines (200–400 °C). Therefore, the catalyst is needed to reduce the soot combustion temperature, promote the passive regeneration of DPF, and reduce the pressure of the filter^5^.

Currently, commercial soot combustion catalysts contain about 0.75 wt% of platinum, which is accounted for one third of the total cost of the filter^6^. Therefore, a large number of non-noble metal catalysts (such as transition metal, alkali metal, alkaline earth metal, perovskite, cerium composite oxide catalysts, etc.) have been extensively studied in order to replace platinum in DPF^7–13^. Among the different types of soot oxidation catalysts, cerium manganese composite oxide catalysts are considered as potential substitutes for Pt/Al_2_O_3_ catalyst which has been commercialized owing to their good oxidation activity^6^.

The rare earth element cerium has excellent oxygen storage/release capacity due to its unique 4f electron layer structure. According to the “reactive oxygen species mechanism”, the reactive oxygen species released by CeO_2_ is very conducive to soot oxidation due to the good reversible conversion efficiency of Ce^4+^/Ce^3+^^14,15^. As the 3d orbital is not filled, transition metal manganese possesses many valence states, and the transformation of different valence states will form oxygen vacancies during the catalytic soot combustion process, thus showing high catalytic activity^16^. Cerium manganese composite oxide catalysts have been widely studied because they can combine the advantages of the above two catalysts and further improve the catalytic activity of soot oxidation^6^.

From the current research on the catalytic soot combustion of cerium-manganese bimetallic catalysts in O_2_ atmosphere, it is mainly focused on improving the intrinsic properties of catalysts (increasing the amount of reactive oxygen species) and changing the morphology of catalysts so as to promote the contact ability between catalysts and soot. Mukherjee et al.^17^ studied the effects of different doped elements (rare earth metals and transition metals Zr, Hf, Fe, Mn, Pr and La) on soot combustion of CeO_2_ catalyst and found that the catalyst doped with Mn exhibited highest concentration of surface adsorbed oxygen species and most loosely bound lattice oxygen among all the materials, thus showing the best soot oxidation activity. Liang et al.^18^ found that under loose contact condition, the soot catalytic combustion activity of MnO_x_–CeO_2_ was higher than that of CuO_x_–CeO_2_ because adding Mn^x+^ into CeO_2_ lattice could promote the generation of more oxygen vacancies, thus promoting the adsorption of oxygen on the surface. He et al.^19^ compared Ce_0.5_Zr_0.5_O_2_ catalyst modified with different transition metals Mn, Fe and Co, and found that the soot catalytic activity of Ce_0.5_Zr_0.5_O_2_ catalyst doped with Mn or Co was superior to that doped with Fe due to the increased reactive oxygen species and lattice oxygen mobility of the catalyst. Wang et al.^20^ synthesized Mn_x_Ce_1-x_O_2_ solid solutions within mesoporous nanosheets by hydrothermal method. The catalyst had excellent soot combustion performance mainly due to its unique mesoporous nanosheet-shaped feature, high-valence Mn species, abundant reactive oxygen species and high redox performance. Zhao et al.^21^ prepared a series of MnO_x_–CeO_2_ composites and found that the catalytic activity of soot was the best when Mn/(Mn + Ce) was 20 at%. This was because the porous structure of the catalyst was similar to the size of soot particles, which was conducive to the contact between the catalyst and soot.

In addition, according to the research results of Kang et al.^22^, the solubility of Mn^x+^ in cerium manganese solid solution has a certain limit, beyond which, the crystal cell parameters of cerium manganese solid solution will no longer shrink, and the excessive manganese would exist on the surface of the solid solution in a highly dispersed state. It is well known that manganese oxides with high surface dispersion are difficult to be detected by XRD^23–25^. In our previous study^26^, we found that these surface manganese oxides could not only exhibit good reduction performance at low temperature, but also facilitate the contact between soot and catalyst. Therefore, if more surface manganese oxides can be provided, the catalytic combustion of soot is promoted.

Li et al.^27^ found that the two series of CeO_2_-based and Fe_2_O_3_-supported oxides prepared by hydrothermal method have more oxygen vacancies and more small CeO_2_ nanoparticles on Fe_2_O_3_, respectively. The concentration of oxygen vacancy is mainly dependent on the content of iron in ceria lattice, and the formation of surface Fe–O–Ce species is dependent on the particle size of surface CeO_2_. In that study, the effects of oxygen vacancy and surface Fe–O–Ce species on catalytic soot combustion were compared, and it was found that high concentration of oxygen vacancy was more beneficial to soot catalytic combustion. However, the comparison of the effects of oxygen vacancy in cerium manganese solid solution and highly dispersed MnO_*x*_ on catalytic soot combustion has not been reported.

In this experiment, we found that the cerium manganese catalysts prepared with Na-containing precipitants (NaOH and Na_2_CO_3_) had more oxygen vacancies due to the entrance of Na into the CeO_2_ lattice (no dispersed MnO_*x*_ on the surface due to the formation of Na_0.7_Mn_0.2_O_5_), while the surface of cerium manganese catalysts prepared with Na-free precipitants ((NH_4_)_2_CO_3_ and NH_3_·H_2_O) contained many highly dispersed MnO_*x*_. Therefore, two series of cerium manganese catalysts were prepared with or without Na precipitants, and the effects of oxygen vacancy and surface MnO_*x*_ on soot catalytic combustion were investigated. The reasons for the differences in soot combustion activity caused by oxygen vacancy and surface MnO_**x**_ on different series of catalysts were revealed by characterizations of X-ray diffraction (XRD), N_2_ adsorption/desorption, Scanning electron microscope (SEM), X-ray photoelectron spectroscopy (XPS), Raman spectra, H_2_ temperature programmed reduction (H_2_-TPR), O_2_ temperature programmed desorption (O_2_-TPD), soot temperature programmed reduction mass spectrum (soot-TPR-MS) and in situ IR.

## Results

### Catalytic performances for soot combustion

**Figure 1:**
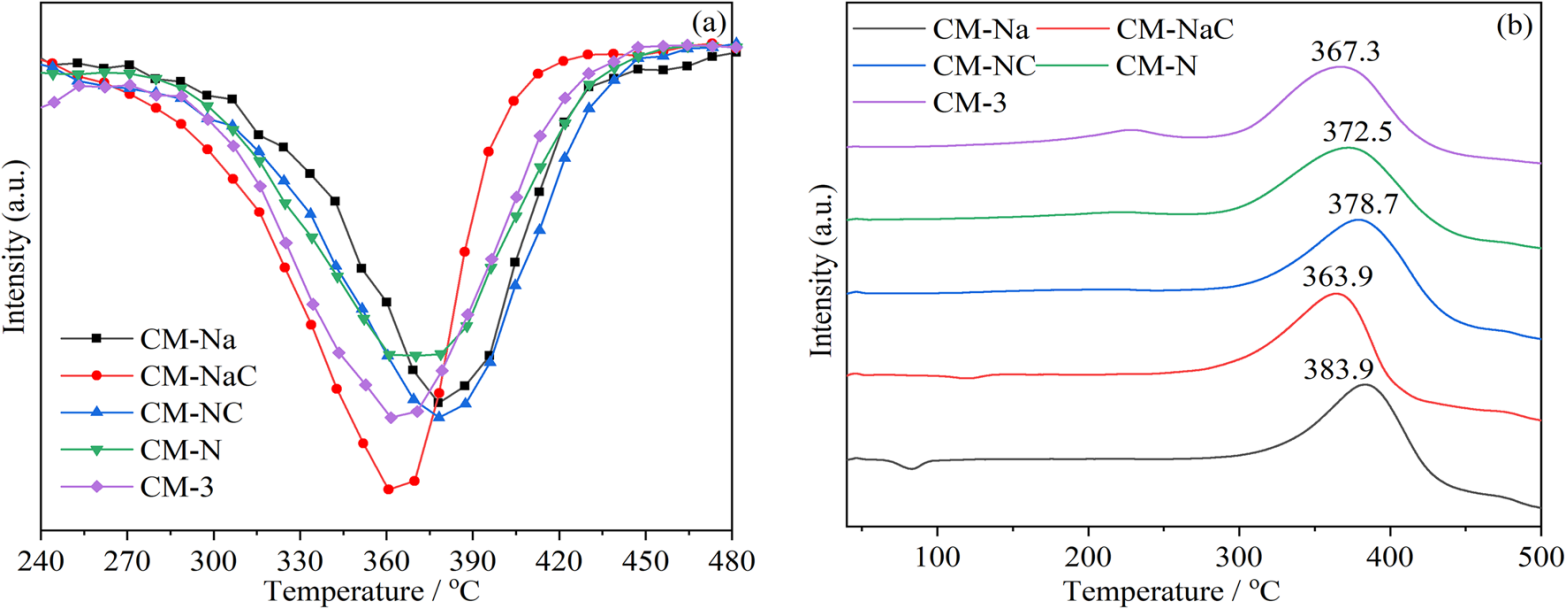
Soot catalytic activities of the catalysts: (**a**) DTG curves; (**b**) DSC curves. (under the tight contact condition).

Figure 1 presents the diferential thermogravimetric (DTG) curves and Differential Scanning Calorimetry (DSC) curves of cerium manganese catalysts prepared with different precipitants for soot combustion under tight contact condition. (The activity results under loose contact condition are shown in Fig. S1) The T_*m*_ of soot combustion without catalyst is as high as 662 °C. The results show that the addition of catalysts significantly reduces the soot combustion temperature. For Na-containing catalysts, the activity of CM-NaC is superior to that of CM-Na, and for Na-free catalysts, the order of activity is CM-3 > CM-N > CM-NC. The T_*m*_ of CM-NaC and CM-3 for soot combustion are 363.9 °C and 367.3 °C, respectively, which are 298.1 °C and 294.7 °C lower than that without catalyst. The activity of CM-NaC is better than that of CM-3, which is more obvious under the loose contact condition (Fig. S1).

**Figure 2:**
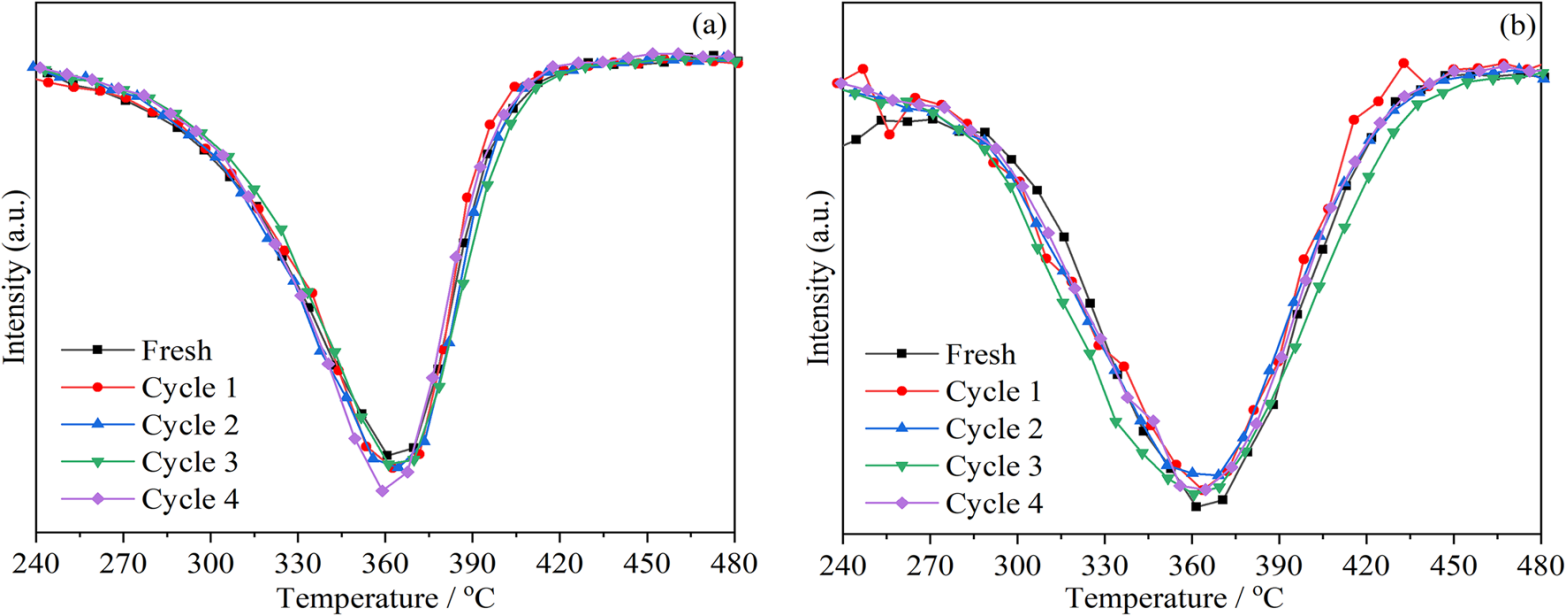
The stability test of the catalysts: (**a**) CM-NaC; (**b**) CM-3. (under the tight contact condition).

Under the same reaction conditions, CM-NaC and CM-3 with good activity were selected for 4 cycle tests to further determine the stability of this series of cerium manganese catalysts. It can be seen from Fig. 2 that the activities of the catalysts used for 4 times are almost the same, and the repeated use hardly causes any deactivation of the catalysts, indicating good stability, which can meet the reuse needs of the catalyst in the process of application.

In order to exclude the effect of the release of CO_2_ and H_2_O from the physical adsorption of the catalysts or CO_2_ released by the catalyst itself on the activity, the release of CO_2_, CO and H_2_O from the catalyst and catalyst + soot during the temperature programmed process was detected by mass spectrum. The related analyses are shown in Fig. S2.

### Structural and textural properties

**Figure 3:**
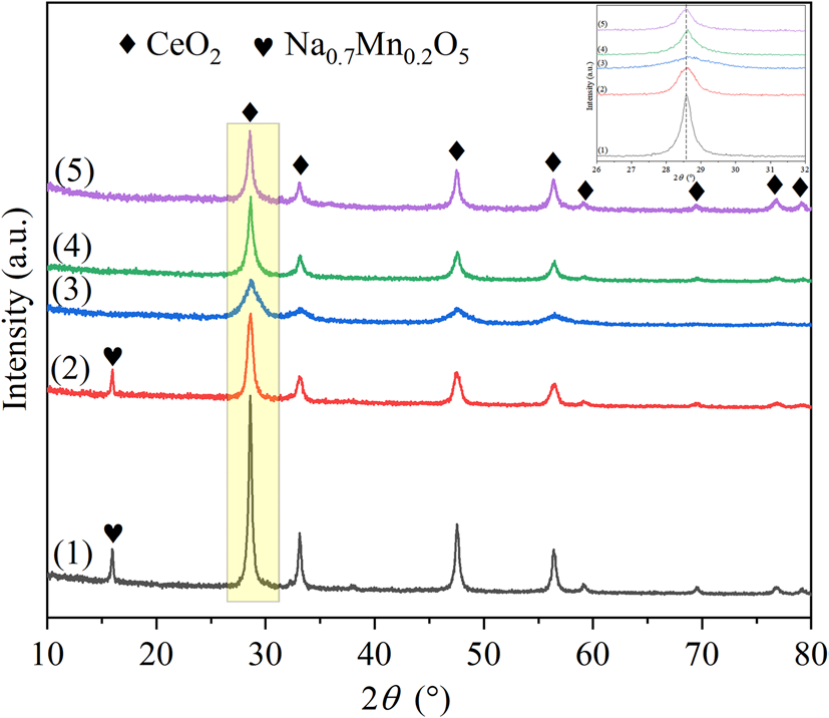
XRD patterns of cerium manganese catalysts: (1) CM-Na; (2) CM-NaC; (3) CM-NC; (4) CM-N; (5) CM-3.

The XRD patterns of the CM-Na, CM-NaC, CM-NC, CM-N, CM-3 catalysts are shown in Fig. 3. For Na-free catalysts, the diffraction patterns are identified as the cubic fluorite-like structure of CeO_2_ (JCPDS #34-0394). The diffraction peaks are at around 28.5°, 33.1°, 47.5°, 56.3°, 59.1°, 69.4°, 76.7° and 79.1°, respectively, which represent the 111, 200, 220, 311, 222, 400, 331 and 420 crystal planes of cubic CeO_2_, respectively. No diffraction peaks of any manganese oxides were detected, which may be because that the manganese has entered into the CeO_2_ lattice and cerium manganese solid solution is formed, or because that the manganese oxides are highly dispersed on the surface of the cerium manganese solid solution and cannot be detected by XRD technique^23–25^. According to literature report^26^, the solubility of manganese in CeO_2_ lattice in cerium manganese catalyst is smaller than 36%, so it can be inferred that most of the manganese species has entered into the CeO_2_ lattice to form solid solution, with only a small proportion of manganese oxide dispersed on the surface of the cerium manganese solid solution.

In the case of the Na-containing catalysts, in addition to the diffraction peaks of cubic fluorite-like structure of CeO_2_, the diffraction peak of Na_0.7_Mn_0.2_O_5_ (JCPDS #27-0751) can also be detected. The dominant diffraction peak is located at around 15.9°, corresponding to 002 crystal plane. The diffraction peak intensities of other crystal planes are only 5–20% of that of the 002 crystal plane, making them difficult to be observed. This phenomenon indicates that Na_0.7_Mn_0.2_O_5_ will be formed when Na-containing alkali was used as precipitant to synthesize cerium manganese catalyst, thus more Mn will be separated out from the cerium manganese solid solution and the structure of the solid solution will be destroyed.

By comparing the changes of lattice parameters (Table 1), it can be found that the lattice parameter of CM-NC is the smallest. Since the ionic radii of Ce^4+^ (0.094 nm) and Ce^3+^ (0.114 nm) are larger than those of Mn^n+^ (Mn^4+^  = 0.054 nm, Mn^3+^  = 0.066 nm and Mn^2+^  = 0.080 nm)^28,29^, it indicates that the amount of manganese entering into the CeO_2_ lattice in CM-NC is the largest, and the diffraction peak of its 111 crystal plane is also right-shifted to the largest extent. The order of cell parameters of the Na-free catalysts is CM-NC < CM-N < CM-3, so the amount of manganese entering into the CeO_2_ lattice in CM-3 catalyst is the smallest, and the surface fine manganese oxide particles of the catalyst are the most.

**Table 1:** Structural and textural properties of cerium manganese catalysts.

Catalyst	Lattice constant α (nm)	d111 (nm)	S_BET_ (m^2^/g)	V (ml/g)	Average pore diameter (nm)
CM-Na	0.5407	19.3	6	0.028	6.84
CM-NaC	0.5398	13.2	9	0.042	34.01
CM-NC	0.5380	6.0	76	0.114	6.80
CM-N	0.5399	12.8	20	0.071	11.15
CM-3	0.5411	13.8	27	0.085	6.76

In addition, by comparing the diffraction peak intensities (Fig. 3), it can be found that the diffraction peak intensity for the cubic fluorite-like structure of the CM-Na catalyst is much higher than those of the other four catalysts, and the diffraction peak intensity of CM-NC catalyst is the lowest. This indicates that CM-Na has the highest degree of crystallization with the largest crystallite size, while CM-NC has the lowest degree of crystallization with the smallest crystallite size, which is consistent with the change of crystallite size of d (111) crystal plane calculated by Scherrer’s equation in Table 1. This is contrary to the change order of specific surface area, which indicates that for Na-containing precipitants, compared with NaOH, Na_2_CO_3_ is more beneficial to restrain the growth of crystallite and promote the increase of specific surface area. For Na-free precipitants, more smaller grains can be produced with (NH_4_)_2_CO_3_ than with NH_3_·H_2_O.

**Figure 4:**
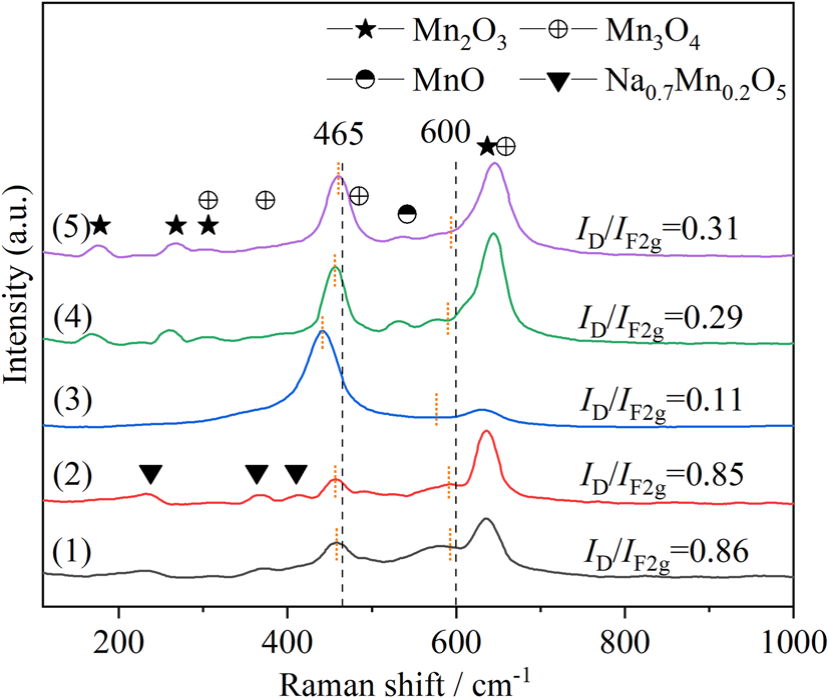
Raman spectra of cerium manganese catalysts: (1) CM-Na; (2) CM-NaC; (3) CM-NC; (4) CM-N; (5) CM-3.

XRD characterization is difficult to detect highly dispersed or MnO_*x*_ phase with low concentration in the study of CeO_2_–MnO_*x*_ system, and according to literature reports^30–32^, the amount of oxygen vacancies plays an important role in soot catalytic combustion, so the Raman characterization which is more sensitive to the lattice vibration of oxygen is used to further analyze the structure of catalyst. All the catalysts exhibit Raman band at around 465 cm^−1^ (Fig. 4), which is characteristic of the symmetric stretching vibrations (F_2g_) in the cubic fluorite CeO_2_^33^. The F_2g_ peaks of all catalysts slightly shift to the left, which can be attributed to the lattice distortion of CeO_2_ and the formation of solid solution caused by the incorporation of smaller Mn^x+^^34^. The F_2g_ peak of CM-NC shifts to the largest extent, indicating that the amount of Mn^x+^ entered into the CeO_2_ lattice is the largest, which is consistent with the XRD analysis results. Therefore, the peak intensity of corresponding MnO_*x*_ in the Raman spectrum of CM-NC is the lowest. For CM-3 and CM-N, signals of Mn_3_O_4_, Mn_2_O_3_ and MnO can be detected^35^, which well confirms that manganese does not fully enter into the CeO_2_ lattice, but is partially dispersed on the surface of the solid solution in the form of MnO_*x*_. For the Na-containing catalysts, peaks at 236, 365 and 412 cm^−1^ are also detected, which could be inferred by XRD to be Na_0.7_Mn_0.2_O_5_ peaks.

The peak at around 600 cm^−1^ is considered to be related to oxygen vacancy^36^, and its intensity is indicated by* I*_D_. The amount of oxygen vacancies on cerium-based catalysts can be evaluated by *I*_D_/*I*_F2g_, the greater the value, the more oxygen vacancies. Similar with the peak at 465 cm^−1^, the peak at 600 cm^−1^ is also shifted after Mn doping. The movements of 600 cm^−1^ peaks are marked in the figure according to the distance of the movements of 465 cm^−1^ peaks. The values of *I*_D_/*I*_F2g_ of the Na-containing catalysts are much higher than those of the Na-free catalysts (Fig. 4), indicating that the application of Na-containing precipitants is beneficial to the generation of oxygen vacancies, which is beneficial to the adsorption and activation of gaseous oxygen. In addition, the amount of oxygen vacancies produced by using NH_3_·H_2_O as precipitant is larger than that produced by using (NH_4_)_2_CO_3_ as precipitant.

According to the specific surface areas calculated by Multi-Point BET method (Table 1), the CM-NaC catalyst with the excellent soot catalytic combustion activity has a small specific surface area. This is because it has been reported^19,23,37^ that the correlation between specific surface and soot combustion activity is very low. In addition, there is an interesting discovery that using Na_2_CO_3_ as precipitant can expand the average pore diameter of the catalyst to larger than 30 nm (larger than the average size of soot 25 nm), which will facilitate the soot to enter the pores of the catalyst and promote the soot catalytic combustion. However, the average pore diameters of the catalysts prepared with other precipitants are smaller than the average size of soot, so it is difficult for soot to enter the pores of these catalysts.

Based on the above analysis about the structural and textural properties, it can be concluded that: when different precipitants were used, the content of manganese in the cerium manganese solid solution is not consistent. (1) For the Na-free catalysts, the cell parameter of CM-3 is the largest, so the amount of MnO_*x*_ on its surface is the largest, which is followed by CM-N and CM-NC. Raman was used to further demonstrate the presence of highly dispersed manganese oxides on the surface of Na-free catalysts. (2) For the Na-containing catalysts, the formation of Na_0.7_Mn_0.2_O_5_ in the precipitation process leads to the disappearance of MnO_*x*_ on the surface of the catalyst and the formation of a large number of oxygen vacancies. Na_2_CO_3_ is more beneficial to inhibit the growth of microcrystals and promote the increase of pore diameter than NaOH. There is an interesting discovery that when Na_2_CO_3_ was used as precipitant, the average pore diameter of the catalyst can be enlarged to above 30 nm (larger than the average size of soot 25 nm), which will help soot enter the pore of the catalyst and promote soot catalytic combustion.

### SEM–EDS

**Figure 5:**
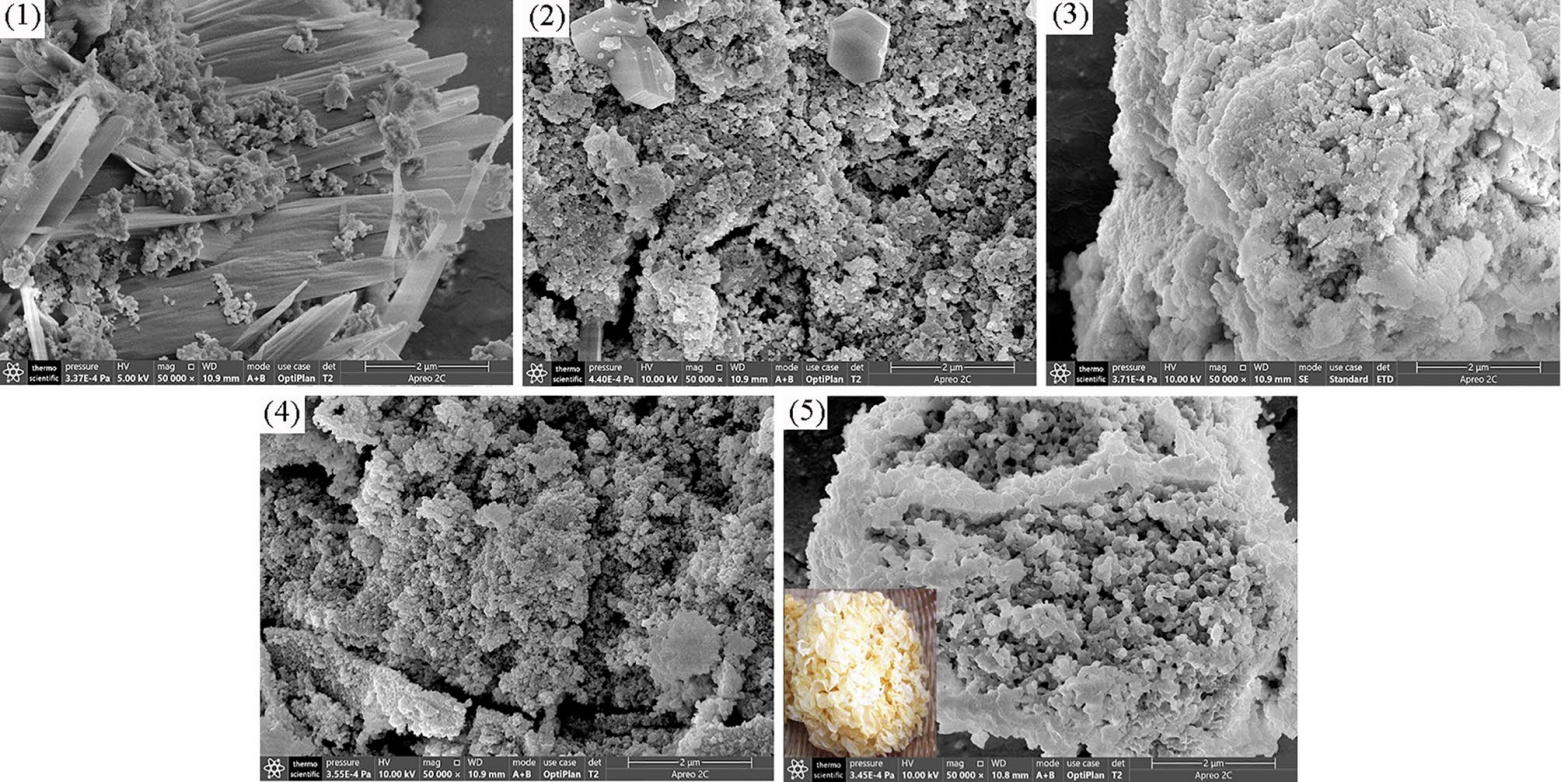
SEM images of cerium manganese catalysts: (1) CM-Na; (2) CM-NaC; (3) CM-NC; (4) CM-N; (5) CM-3.

Figure 5 shows the morphology of cerium manganese catalysts prepared with different precipitants. If (NH_4_)_2_CO_3_ was used as precipitant, the shape of the catalyst is similar to tremella. If NH_3_·H_2_O was used as precipitant, a catalyst consisting of many small particles is obtained. If both (NH_4_)_2_CO_3_ and NH_3_·H_2_O were used as precipitants, the shape of the catalyst is between those of the above two catalysts, like tremella with many small particles wrapped in the middle. The morphology, which combines the advantages of the two catalysts, is conducive to the full contact between soot and catalyst, which is more conducive to the soot combustion. If NaOH or Na_2_CO_3_ was used as precipitant, the catalysts are flake and granular. Combined with the XRD results, it is speculated that the flake and granular parts may be composed of different phases. The related analyses are shown in Fig. S3 and Table S1.

### H_2_-TPR characterization

It has been reported in many literatures that the good redox properties of catalysts have a positive effect on the soot catalytic combustion^20,38^. In general, the mutual doping of MnO_*x*_ and CeO_2_ can improve the redox performance of a single oxide^31,39^. The peak α starting from the low temperature region of about 150 °C can be attributed to the reduction of highly dispersed and easily reduced MnO_*x*_ on the surface^40,41^. This low temperature reduction peak can be clearly seen on CM-NC, CM-N and CM-3. However, for CM-Na and CM-NaC, this low temperature peak can hardly be observed, especially for CM-NaC, the initial peak of which begins at 350 °C. According to Raman analysis, the phase of MnO_*x*_ can be found on CM-NC, CM-N and CM-3. Therefore, it can be inferred that MnO_*x*_ are dispersed in the form of fine particles on the surface of the solid solution, which can also be confirmed by the XPS results that the content of surface manganese is higher than the theoretical value. As for the Na-containing catalysts, according to the XRD results, the phase with CeO_2_ cubic fluorite-like structure and Na_0.7_Mn_0.2_O_5_ can be detected, no phase of MnO_*x*_ appears. The MnO_*x*_ on the surface of cerium manganese solid solution disappear due to the formation of Na_0.7_Mn_0.2_O_5_, resulting in the disappearance of low temperature reduction peak α. The reduction peaks β, γ and δ between 300 and 700 °C can be attributed to the reduction of Mn^4+^  → Mn^3+^  → Mn^2+^ and Ce^4+^  → Ce^3+^on the surface^39,42^. The reduction peak ε at about 800 °C belongs to the reduction of Ce^4+^ in the bulk^43^. However, the temperature of soot combustion is lower than 800 °C, so the reduction of Ce^4+^ in the bulk has no effect on the soot catalytic combustion.

The statistical results of hydrogen consumption of peaks α + β + γ + δ are shown in Table 2. CM-NaC has the highest hydrogen consumption, which is mainly due to its larger amount of oxygen vacancies according to the Raman analysis, which may be the reason for its good activity. But what's interesting is that the hydrogen consumption of CM-Na ranks second, but it's activity is bad. Moreover, for the Na-free catalysts, the activity does not correspond well to the hydrogen consumption below 700 °C. Therefore, for all catalysts in this series, the hydrogen consumption below 700 °C is not directly related to the soot combustion activity.

**Table 2:** Reducing property of cerium manganese catalysts.

Catalyst	H_2_ consumption(μmol/g)			S_max_^a^	T_α_ (°C)	T_β_ (°C)
	α + β + γ + δ	α	β			
CM-Na	2813	–	916	–	–	335
CM-NaC	3621	–	1470	–	–	482
CM-NC	2070	232	760	0.159	243	322
CM-N	1992	214	697	0.158	240	298
CM-3	2250	272	592	0.286	233	275

^a^The maximum slope of the α reduction peak.

**Figure 6:**
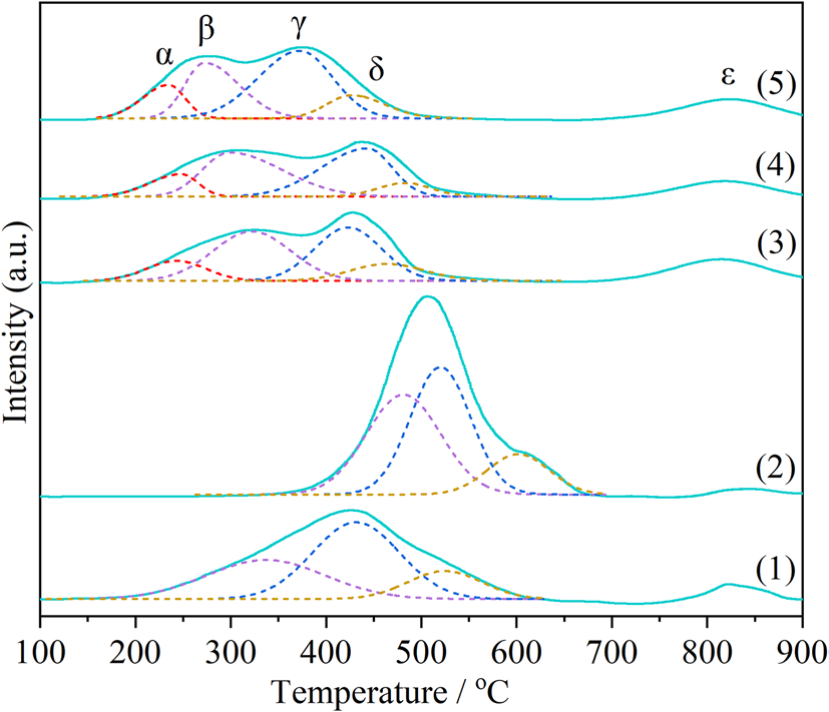
H_2_-TPR profiles of cerium manganese catalysts: (1) CM-Na; (2) CM-NaC; (3) CM-NC; (4) CM-N; (5) CM-3.

According to literature reports^37,44^, the complete combustion temperature of soot is lower than 500 °C due to the action of catalyst, so the hydrogen consumption performance at low temperature is the key factor to determine the activity. Therefore, the low temperature hydrogen consumption rate, the low temperature reduction peak temperature and the low temperature hydrogen consumption amount of the catalyst should also be considered. The maximum slope of the reduction peak α (S_max_) can represent the maximum initial hydrogen consumption rate of the peak. It can be speculated that the higher the S_max_ is, the lower the reduction peak temperature is, the greater the hydrogen consumption at low temperature is, the better the soot combustion activity can be obtained. The initial peak α is the reduction peak of highly dispersed MnO_*x*_ on the surface. Highly dispersed MnO_*x*_ can increase the number of interfaces between cerium manganese solid solution and MnO_*x*_, thus increasing the contact probability of soot and catalyst, and improving the catalytic activity of soot. In addition, peak β, which is slightly higher than peak α, is also in a relatively lower temperature region, so its peak temperature and hydrogen consumption also play a role in soot catalytic combustion activity. T_α_ of CM-3 is the lowest, S_max_ is the largest and the hydrogen consumption of peak α is the highest, followed by CM-N and CM-NC, which is consistent with the order of the activity. As can be seen from Fig. 6, those of the Na-containing catalysts are located at relatively higher temperatures. The reduction peak temperature of CM-NaC is the highest (506 °C), indicating that the low temperature reduction performance of CM-NaC is poor. Since there are no dispersed manganese oxide species on the surface of the Na-containing catalyst, the key factors determining its activity will be further discussed in the subsequent characterization analysis.

In conclusion, It is found that the low temperature hydrogen consumption rate, the low temperature reduction peak temperature and the amount of low temperature hydrogen consumption of the catalyst are the key factors to determine the activity of the Na-free catalysts. As for the Na-containing catalysts, other factors such as the desorption performance of reactive oxygen species should be considered.

### XPS spectra

**Figure 7:**
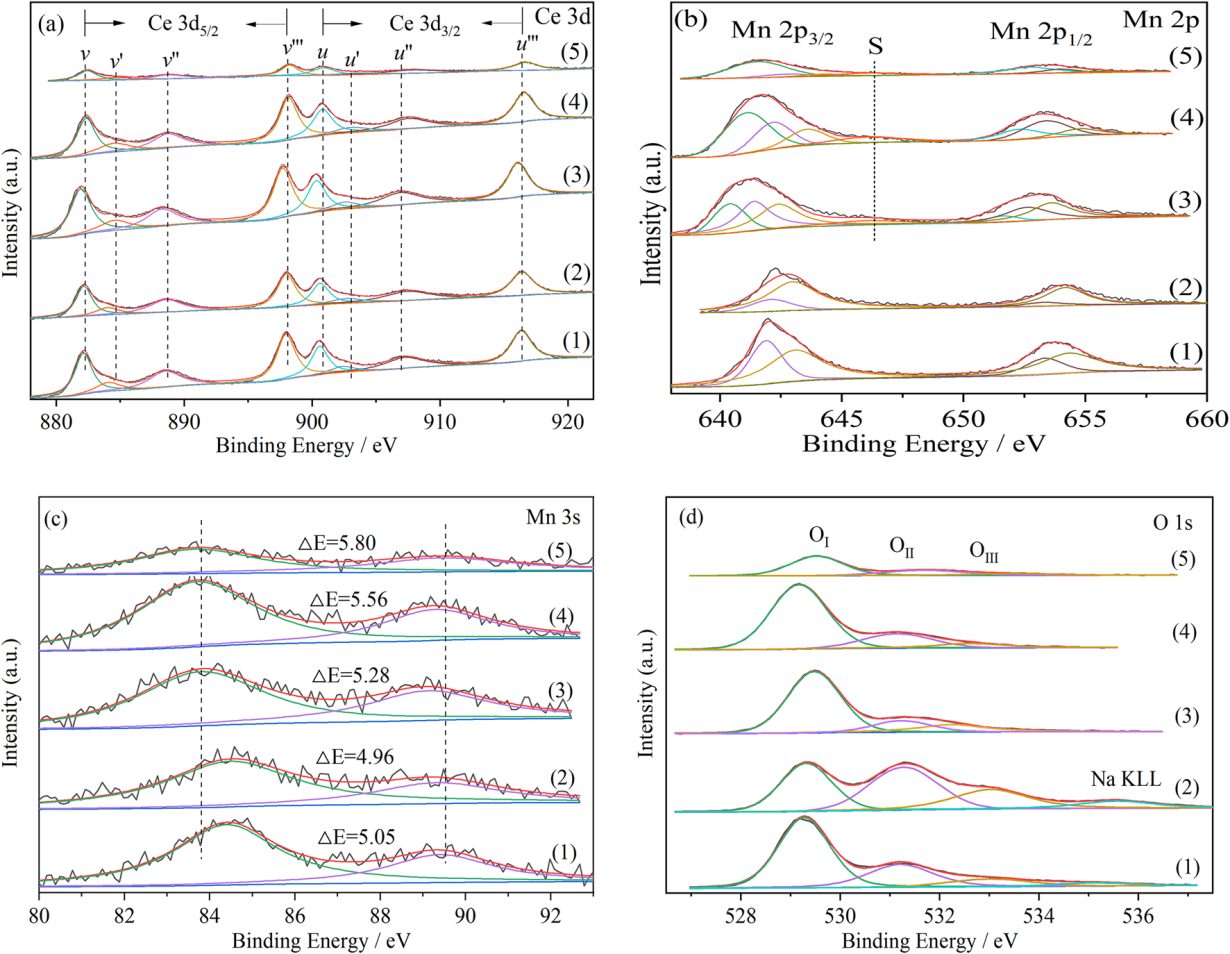
XPS spectra of all cerium manganese catalysts: (**a**) Ce 3d; (**b**) Mn 2p; (**c**) Mn 3s; (**d**) O1s. (1) CM-Na; (2) CM-NaC; (3) CM-NC; (4) CM-N; (5) CM-3.

The elemental compositions on the surface of Na-containing and Na-free catalysts were analyzed by XPS, and the results are shown in Fig. 7 and listed in Table 3. The Ce 3d XPS spectra include two spin–orbit states, 3d_3/2_ (labeled with “*u*”) and 3d_5/2_ (labeled with “*v*”). The peaks denoted as *v*, *v''*, *v'''*, *u*, *u''* and *u'''* are characteristic of Ce^4+^ ions, the other peaks marked as v*'* and u*'* are assigned to Ce^3+^ ions^27,28^. The existence of Ce^3+^ is generally believed to be closely related to the oxygen vacancies and active oxygen^45,46^. The relative content of Ce^3+^ can be calculated according to the area summation ratio of peak *v'* and peak *u'*, and the results are also listed in Table 3. It can be seen that for the Na-free catalysts, the Ce^3+^ content of CM-3 is the highest, so it has the largest amount of oxygen vacancies, which is consistent with the results of Raman characterization. However, the Ce^3+^ contents of the Na-containing catalysts are inconsistent with the content of oxygen vacancies observed from the Raman result, and the reason will be further analyzed by O 1 s.

**Table 3:** Surface elemental analysis derived from XPS.

Catalyst	Ce^3+^ (at%)	O_II_ (at%)	ΔE_s_ (eV)	Average oxidation state (eV)	Mn^2+^ (at%)	Mn^3+^ (at%)	Mn^4+^ (at%)	Mn/(Mn + Ce) atomic ratio
CM-Na	9.06	27.40	5.05	3.64	–-	36.04	63.96	–-
CM-NaC	10.05	39.58	4.96	3.80	–-	19.82	80.18	–-
CM-NC	10.29	14.48	5.28	3.22	19.29	38.91	41.80	0.42
CM-N	11.59	19.14	5.56	2.71	46.49	34.06	19.45	0.48
CM-3	13.07	24.08	5.80	2.27	73.56	17.71	8.73	0.51

The spectra of the Mn 2p region in Fig. 7b show two spin–orbit states with binding energies located in the ranges of 640.0–650.0 eV (Mn 2p_3/2_) and 650.0–660.0 eV (Mn 2p_1/2_) respectively. The ΔE of the binding energies (BE) of Mn 2p_3/2_ and Mn 2p_1/2_ is approximately 11.2 eV. The two peaks with higher binding energies at approximately 643.6 eV and 654.8 eV are associated with characteristic Mn^4+^ cations, and the two peaks with binding energies at around 642.2 eV and 653.3 eV are linked to typical Mn^3+^ cations, while other two small peaks with lower binding energies at about 641.0 eV and 652.3 eV are assigned to Mn^2+^ cations^47^. The satellite peak of MnO at around 646 eV is also observed on CM-NC, CM-N and CM-3^48^. Since the distance of the twin peaks in the Mn 3s spectra (ΔE_s_) decreases monotonically with the increase of the average oxidation state, it can be used to assist the determination of the valence state of Mn^48–50^. The ΔE_s_ between the twin peaks in the Mn 3s spectra is 4.96–5.8 eV. The average oxidation states of Mn are estimated to be 2.27–3.8^50^, as listed in Table 3. The average oxidation states of Mn for Na-containing catalysts are higher than those for Na-free catalysts. For the Na-free catalysts, the order of average oxidation state is CM-3 (2.27) < CM-N (2.71) < CM-NC (3.22). It has been reported that in Mn_x_Ce_1-x_O_2_ catalysts, the presence of low-valent Mn^x+^ is usually associated with the generation of oxygen vacancies and surface adsorbed oxygen species^19,20^. Therefore, the contribution of low-valent Mn^x+^ to oxygen vacancies has a certain effect on the Na-free catalysts. However, for the Na-containing catalysts, it can be inferred that there are other factors resulting in the high concentration of oxygen vacancies.

The O 1 s core level spectra of this series of catalysts are displayed in Fig. 7d. For Na-free catalysts, the spectra can be resolved with Gaussian–Lorenz model functions and fitted into three peaks. The peak at lower binding energy situated at 529.2–529.5 eV is attributed to lattice oxygen species (O_I_) and the peak at relatively higher binding energy 531.2–531.7 eV is assigned to surface adsorbed oxygen species (O_II_). In the end, the peak at highest binding energy located at 532.4–533.5 eV is attributed to surface adsorbed carbonate and hydroxyl species (O_III_)^51^. Surface adsorbed oxygen species (O_II_) play an important role in soot catalytic combustion and are called reactive oxygen species^52–54^. It can be seen from Table 3 that the ratio of O_II_ of the Na-free catalysts is consistent with the content of Ce^3+^ and I_D_/IF_2g_ in the Raman spectra, indicating that the order of the content of surface and bulk oxygen vacancies and surface adsorbed oxygen species is CM-3 > CM-N > CM-NC. For Na-containing catalysts, there is another peak located at 535.1–535.6 eV, which belongs to the sodium auger peak (Na KLL) according to Handbook of X-ray Photoelectron Spectroscopy. The contents of O_II_ of the Na-containing catalysts are higher than those of the Na-free catalysts, but the ratios of Ce^3+^ and the low-valent Mn^x+^ of the Na-containing catalysts are not high. Therefore, it can be inferred that the high surface oxygen adsorption and large amount of oxygen vacancies of the Na-containing catalysts are mainly caused by the entrance of Na^+^ into the lattice of the solid solution. The increase of surface active oxygen is beneficial to the transfer of reactive oxygen species from the surface of catalyst to the soot, thus promoting the oxidation of soot.

The Mn/(Mn + Ce) atomic ratios on the surface of Na-free catalysts are compared with the corresponding theoretical values in Table 3. (According to XRD results, phase separation occurs for the Na-containing catalysts, and the depth of XPS test is usually less than 5 nm. The content of manganese on the surface of the catalyst with phase separation may be different due to the location of the test, so the content of manganese on the surface of the Na-containing catalysts is not calculated.) The Mn/(Mn + Ce) atomic ratios on the surface of the Na-free catalysts are higher than the theoretical value, which is indicative of the existence of highly dispersed MnO_*x*_ on these three catalysts. It is worth noting that the surface composition of manganese in these catalysts changes significantly due to the application of different precipitants. The Mn/(Mn + Ce) atomic ratio on the surface of the catalyst prepared by using the combination of (NH_4_)_2_CO_3_ and NH_3_·H_2_O as precipitant is the highest, followed by NH_3_·H_2_O and (NH_4_)_2_CO_3_ alone, which is consistent with the XRD and H_2_-TPR results.

According to the XPS results: (1) for the Na-free catalysts, the change of the content of oxygen vacancies in the Na-free catalysts is consistent with that of Ce^3+^, and low valence state Mn^x+^ also contributes to the formation of oxygen vacancies. Because the content of Ce^3+^ in CM-3 is the highest, the amount of oxygen vacancies is the largest. CM-3 has the highest surface Mn/(Mn + Ce) atomic ratio, which is consistent with XRD and H_2_-TPR results. (2) For the Na-containing catalysts, more surface reactive oxygen species can be generated due to the incorporation of Na^+^ into the solid solution lattice.

### O_2_-TPD characterization

The desorption behavior of oxygen on the catalyst can be measured by O_2_-TPD tests^55^. The oxygen desorption performance of catalyst plays an important role in the catalytic combustion of soot because the combustion reaction of soot is essentially an oxidation reaction. According to literature reports^18^, the oxygen desorption peak of pure ceria will appear at about 900 °C, but the doping of ceria by manganese will make the desorption peak move forward. In this study, a large oxygen desorption peak is concentrated between 350 and 700 °C, which is mainly attributed to the superposition of desorption signals of oxygen species with different degree of catalyst action (O_ad_^−^, O_ad_^2−^ and O_latt_^2−^)^42^.

**Figure 8:**
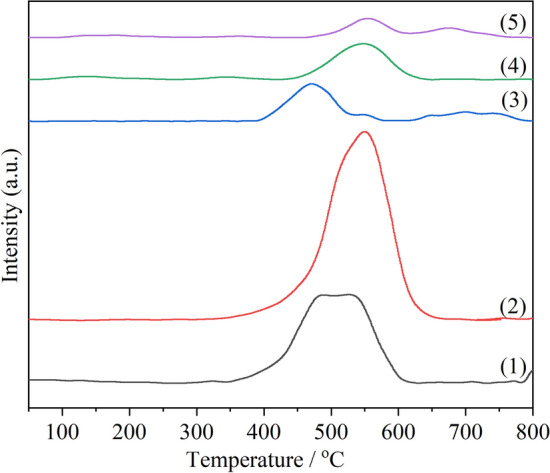
O_2_-TPD profiles of cerium manganese catalysts: (1) CM-Na; (2) CM-NaC; (3) CM-NC; (4) CM-N; (5) CM-3.

As can be seen from Fig. 8, the intensities of the desorption peaks of Na-containing catalysts are much higher than those of Na-free catalysts. That's because for Na-free catalysts, the doping of Mn^x+^ can produce oxygen vacancies in the fluorite-type lattice. For the Na-containing catalysts, Na^+^ and Mn^x+^ enter into CeO_2_ lattice together, resulting in the generation of more oxygen vacancies, and thus desorption of more oxygen species is observed during the temperature-programmed process. The desorption peak of CM-NaC is larger than that of CM-Na, indicating that using Na_2_CO_3_ as precipitant is beneficial to the generation of more oxygen vacancies and the desorption of oxygen species, which is consistent with the results of O 1s XPS. For Na-free catalysts, CM-3 has the highest peak temperature and the smallest peak area, indicating that its oxygen desorption performance is the worst. Therefore, it is further proven that the reason why CM-3 has better catalytic soot combustion activity is independent of the reactive oxygen release capacity of the catalyst.

### Soot-TPR-MS

**Figure 9:**
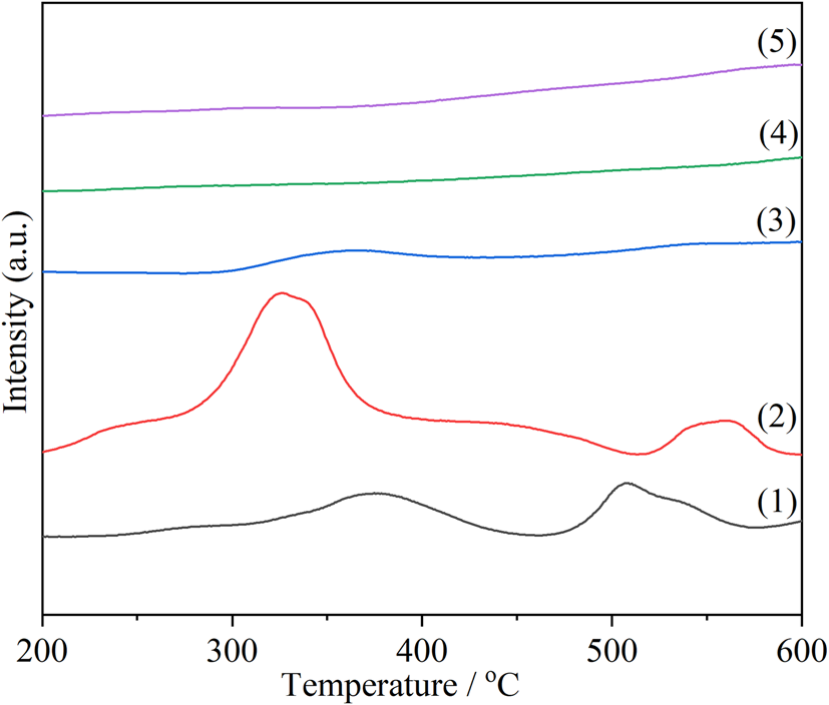
Soot-TPR-MS profiles of cerium manganese catalysts: (1) CM-Na; (2) CM-NaC; (3) CM-NC; (4) CM-N; (5) CM-3.

As the amount of active oxygen and the oxygen mobility are very important for soot catalytic combustion^44,56^, the effect of oxygen species in the catalyst on soot combustion is further studied by soot-TPR. The curves of CO_2_ are shown in Fig. 9. (The contrast curve of CO and CO_2_ production is shown in Fig. S4. Except for CM-NaC, almost no CO production can be observed). It is obvious that the soot reduction peaks in Na-containing catalysts are much larger than that in Na-free catalysts, which indicates that the amount of active oxygen in Na-containing catalysts is much larger than that in Na-free catalysts. In addition, compared with CM-Na and CM-NaC, it can be seen that CM-NaC has more active oxygen and lower reduction peak temperature. Combining Raman and O 1s XPS results, it can be inferred that this is due to the larger number of oxygen vacancies in CM-NaC. The amount and mobility of active oxygen are the key factors to determine the high activity of CM-NaC.

### In situ IR

**Figure 10:**
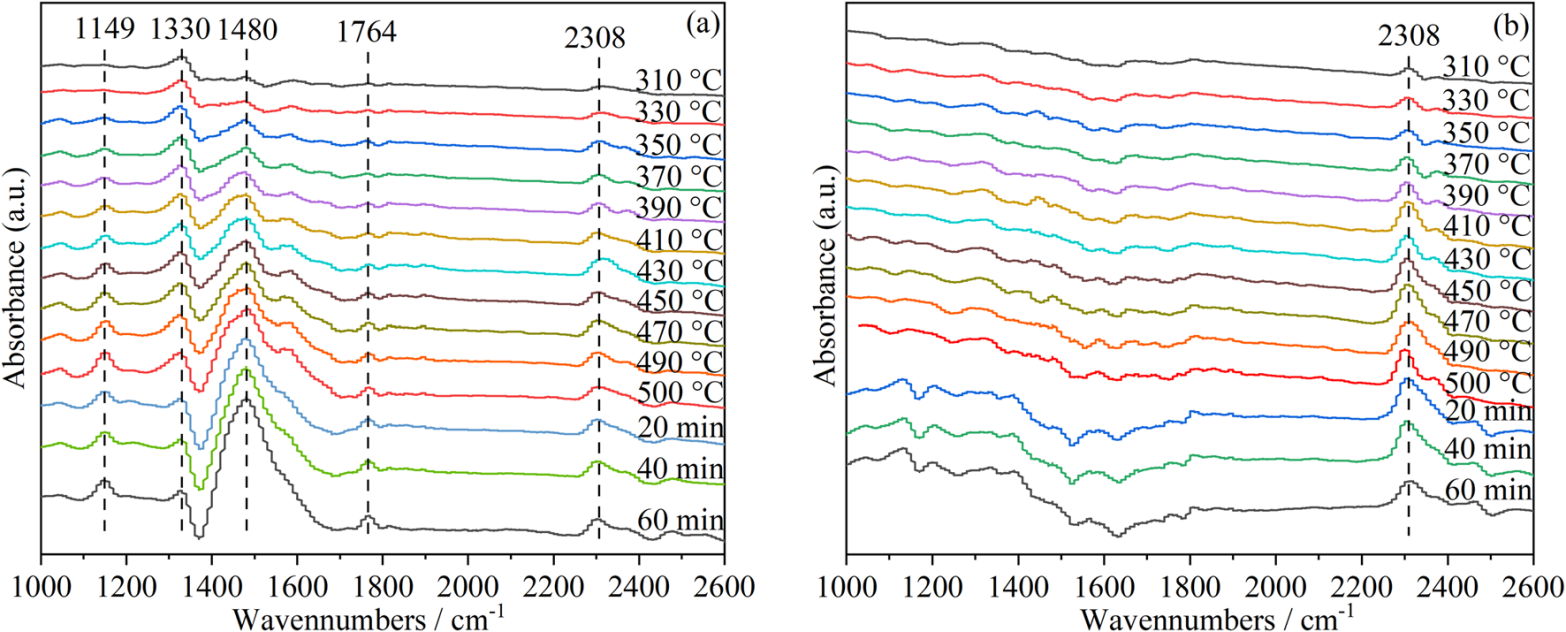
In situ IR spectra for soot catalytic combustion in the flows of 5 vol% O_2_ + He on (**a**) CM-NaC; (**b**) CM-3.

The effects of oxygen vacancy and surface MnO_*x*_ in cerium manganese catalysts on soot combustion were further studied by in situ IR spectra. A series of peaks between 1000 and 1800 cm^−1^ can be observed on CM-NaC with a large number of oxygen vacancies (Fig. 10). The peak at 1149 cm^−1^ can be attributed to bridging bidentate carbonates, while the peaks at 1330 cm^−1^ and 1480 cm^−1^ represent chelating bidentate carbonates, and the peak at 1764 cm^−1^ is assigned to weakly adsorbed CO_2_ species or bridging carbonate^57^. These carbonates are the adsorption of CO_2_ produced during soot combustion on the oxygen vacancies of CM-NaC. The peak at 2308 cm^−1^ is the physical adsorption of CO_2_ produced by soot combustion on the catalyst^58^. An obvious peak of 2308 cm^−1^ can be observed on CM-3, but the peaks in the range of 1000–1800 cm^−1^ are not obvious. This is because there are few oxygen vacancies on CM-3, so it is not easy to adsorb carbonate. This further confirms that the catalytic combustion of soot by CM-NaC mainly depends on the active oxygen species released by oxygen vacancies, while CM-3 with fewer oxygen vacancies mainly depends on good redox performance at low temperature.

## Conclusions

In this study, two series of cerium manganese catalysts were prepared with different precipitants, and the effects of abundant oxygen vacancies and highly dispersed MnO_*x*_ on soot catalytic combustion were investigated. For Na-containing catalysts, a large number of oxygen vacancies are produced due to the destruction of the structure of cerium manganese solid solution by the formation of Na_0.7_Mn_0.2_O_5_, which releases a large amount of active oxygen species in the process of catalytic soot combustion. For the Na-free catalysts, when (NH_4_)_2_CO_3_ and NH_3_·H_2_O were used as precipitants at the same time, the morphology of the catalyst is like tremella with many small particles wrapped in the middle. A large amount of MnO_*x*_ on the surface of the catalyst can increase the number of interfaces between soot and catalyst, which can enhance the contact probability between soot and catalyst, and have good redox performance at low temperature, thus improving the oxidation efficiency of soot. Therefore, a large number of oxygen vacancies in the catalyst and highly dispersed MnO_*x*_ on the surface of the catalyst play an important role in soot catalytic combustion. However, in the catalytic process, the active oxygen released from the oxygen vacancies is more beneficial to the soot catalytic combustion than the surface MnO_*x*_. This phenomenon is more obvious under the loose contact condition, because the catalytic combustion of soot by Na-free catalysts depends more on the contact condition between soot and catalyst. When the contact condition becomes worse, the activity decreases more.

## Methods

### Catalysts preparation

A series of cerium manganese catalysts with Ce:Mn atomic ratio of 6:4 were prepared by co-precipitation using Ce(NO_3_)_3_·6H_2_O (AR grade, Yutai Qixin Chemical, China) and Mn(NO_3_)_2_ (AR grade, Xiya Reagent, China) as starting materials. The precipitants used were NaOH (3 mol·L^−1^), Na_2_CO_3_ (3 mol·L^−1^), (NH_4_)_2_CO_3_ (3 mol·L^−1^), NH_3_·H_2_O (3 mol·L^−1^) and a mixture of (NH_4_)_2_CO_3_ and NH_3_·H_2_O with molar concentration ratio of 3/3, accordingly, the catalysts prepared were abbreviated as CM-Na, CM-NaC, CM-NC, CM-N and CM-3, respectively. The salt solution and the alkali solution were mixed together under continuous stirring, keeping the pH around 8.5–8.8 during this process.. The precipitate slurry was filtered and washed with water. Then the precipitates were dried at 70 °C for 24 h and calcined at 600 °C for 3 h to obtain the prepared catalyst sample. In addition, the catalysts were divided into two groups according to the presence or absence of Na in the precipitant: Na-containing catalysts (CM-Na and CM-NaC) and Na-free catalysts (CM-NC, CM-N and CM-3).

### Characterization

The characterization methods of X-ray diffraction (XRD), N_2_ adsorption–desorption, scanning electron microscopy (SEM), Raman spectroscopy, X-ray photoelectron spectroscopy (XPS), H_2_ temperature-programmed reduction (H_2_-TPR) and O_2_ temperature-programmed desorption (O_2_-TPD), Soot temperature programmed reduction (Soot-TPR) and in situ IR spectra are described in the Supporting information.

### Catalytic activity measurement

The catalytic activity of cerium manganese catalyst for soot combustion was measured by means of TGA/DSC thermogravimetric analyzer (METTLER, Swiss) with Printex-U (Degussa, Germany) used as the model of diesel soot. The soot and the catalyst (weight ratio was 1:10) were carefully ground in a mortar for 10 min to achieve the “tight contact” condition. In order to make the evaluation condition similar to the actual condition, the catalyst and soot were mixed with shovel for 5 min to achieve the “loose contact” condition. The reaction test was carried out with 10% O_2_/N_2_ which was close to the oxygen concentration in diesel exhaust. Activity tests were carried out from 30 to 600 °C under a gas flow rate of 30 ml/min, and the heating rate was maintained at 10 °C/min. T_*m*_ represents the temperature corresponding to the maximum heat release during soot combustion in the DSC diagram and the peak value of the DTG curve (They're the same). The lower the T_*m*_, the easier the combustion of soot and the better the activity of the catalyst. TPO-MS was used to detect the CO_2_, CO and H_2_O produced during the heating process (Supporting information).

## Supplementary Information

Supplementary Information.

## Data Availability

All data included in this study were obtained by contacting the corresponding authors.
