# Supplementary material for: Effect of oxygen vacancy and highly dispersed MnOx on soot combustion in cerium manganese catalyst
Source: Sci Rep. 2023 Feb 28;13:3386. doi: 10.1038/s41598-023-30465-7 (PMC9975190; doi:10.1038/s41598-023-30465-7)
Supplement: Supplementary file 1 — Supplementary Information. [file 41598_2023_30465_MOESM1_ESM.pdf]

Effect of oxygen vacancy and highly dispersed MnO_x on soot combustion in cerium manganese catalyst

Yi Zhu[1,2,*], Zhen Chen[1,2], Hongmei Li[1,2], Quan Wang[1,2], Xingyu Liu[1], You Hu[1], Cuimei Su[1], Rui Duan[1], Shanhu Chen[3], & Li Lan[4,*]

[1] College of Chemistry Biology and Environment, Yuxi Normal University, Yuxi, 653100, China

[2] Institute of Biology and Environmental Engineering, Yuxi Normal University, Yuxi, 653100, China

[3] College of Chemistry and Chemical Engineering, Jiangxi Science and Technology Normal University, Nanchang, 330013, China

[4] College of Materials and Mechatronics, Jiangxi Science and Technology Normal University, Nanchang, 330013 China

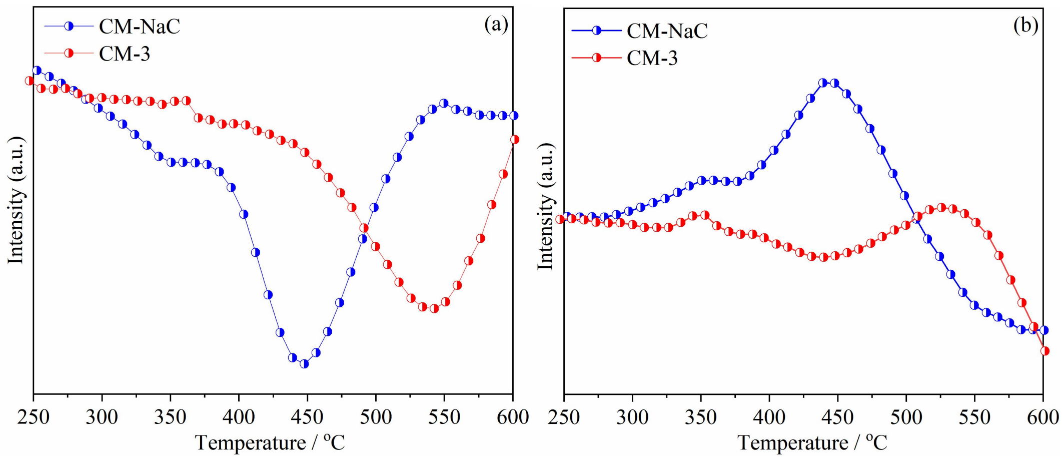

Figure S1. Soot catalytic activities of the catalysts: (a) DTG curves; (b) DSC curves. (under the loose contact condition)

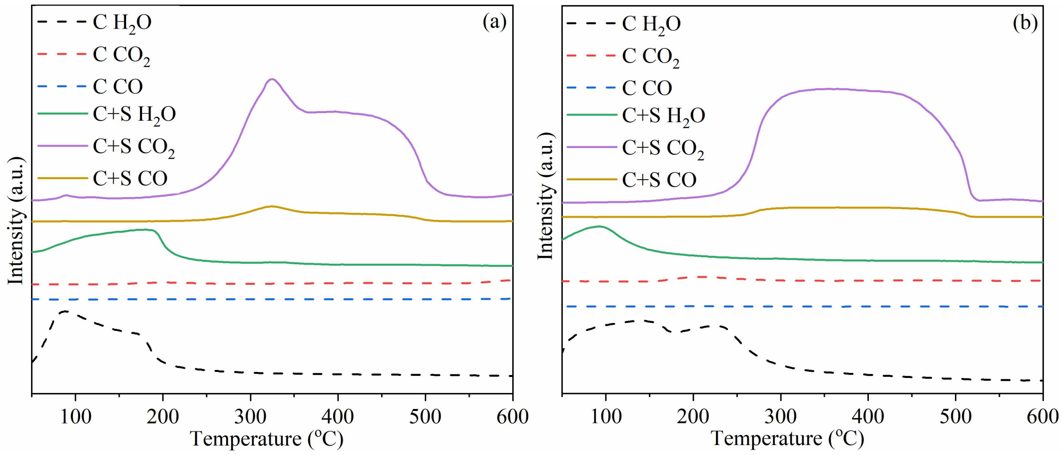

Figure S2. O_2-TPO-MS profiles of catalyst (C) and catalyst +soot (C +S) (under the tight contact condition): (a) CM-NaC; (b) CM-3.

As can be seen from Fig. S2, the catalyst alone and catalyst + soot can release physically adsorbed H_2O before 300 °C. Since the catalysts had been calcined at 600 °C for 3 h, the release of CO_2 and CO is not observed during the heating process, so the peak value of the DTG curve (T_m) between 300 and 600 °C is only caused by soot combustion.

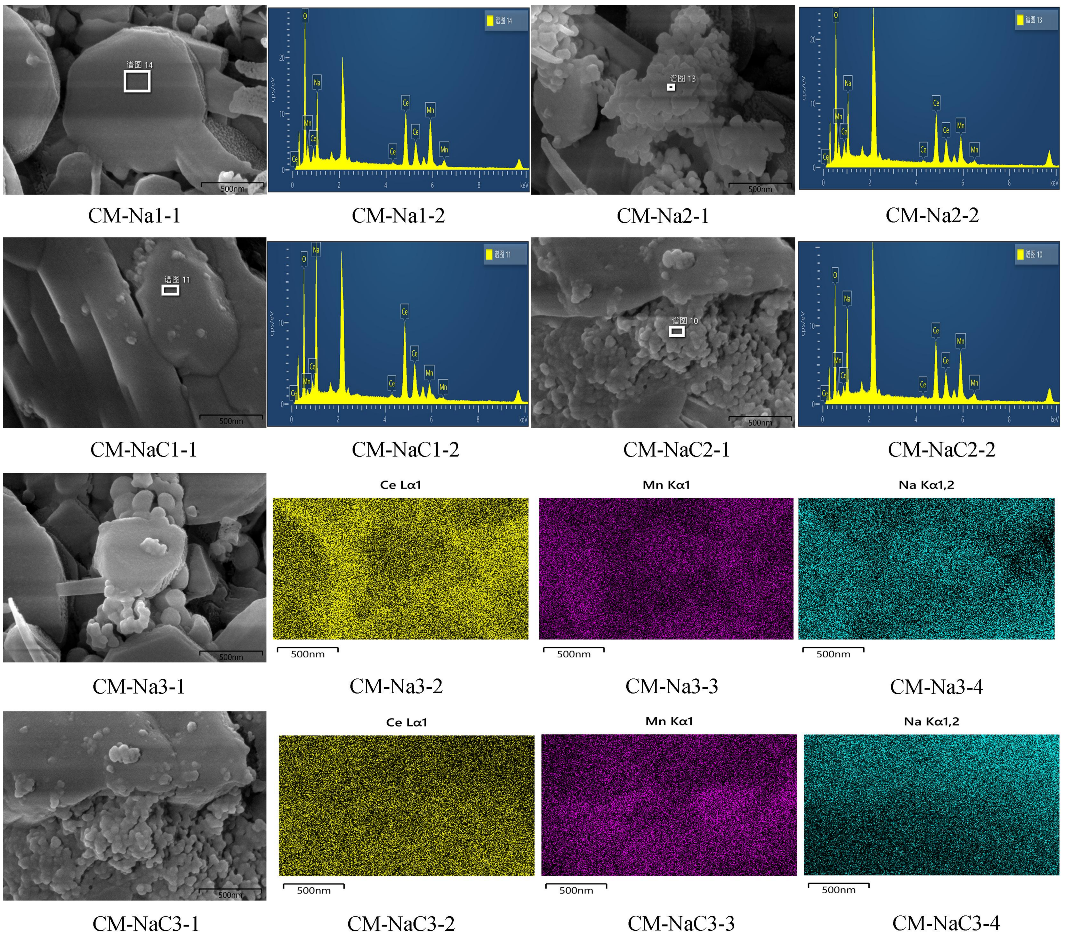

Figure S3. The elemental distributions determined by EDS analysis

Table S1 EDS analysis results of CM-Na and CM-NaC.

catalyst	Ce/(Ce+Mn+Na)	Mn/(Ce+Mn+Na)	Na/(Ce+Mn+Na)
		at%	
CM-Na1	19.85	27.78	52.37
CM-Na2	23.31	19.74	56.95
CM-Na3	27.45	24.81	47.73
CM-NaC1	20.26	6.14	73.60
CM-NaC2	19.10	24.11	56.79
CM-NaC3	15.71	20.64	63.65

If NaOH or Na_2CO_3 was used as precipitant, the catalysts are flake and granular. Combined with the XRD results, it is speculated that the flake and granular parts may be composed of different phases. Therefore, the corresponding EDS and elemental

mapping of Na-containing catalysts are shown in Fig. S3. The atomic ratios of the corresponding elements are shown in Table S1. It's not consistent with the predicted results, Ce, Mn and Na are detected in both granular and flake parts, but the contents of each element are slightly different. The $Na_{0.7}Mn_{0.2}O_5$ phase without Ce detected by XRD does not gather together independently. Therefore, for the Na-containing catalysts, the phase distribution is uniform at the micron/submicron level, but the morphology shows two kinds of structure: flake and granular. By comparing the elemental content of CM-Na and CM-NaC from element mapping, it is found that the Na/(Ce+Mn+Na) content of CM-NaC is higher than that of CM-Na. Combined with the results of the average pore diameter, this phenomenon indicates that more Na^+ has entered into the lattice for CM-NaC, resulting in the larger pore diameter of the catalyst.

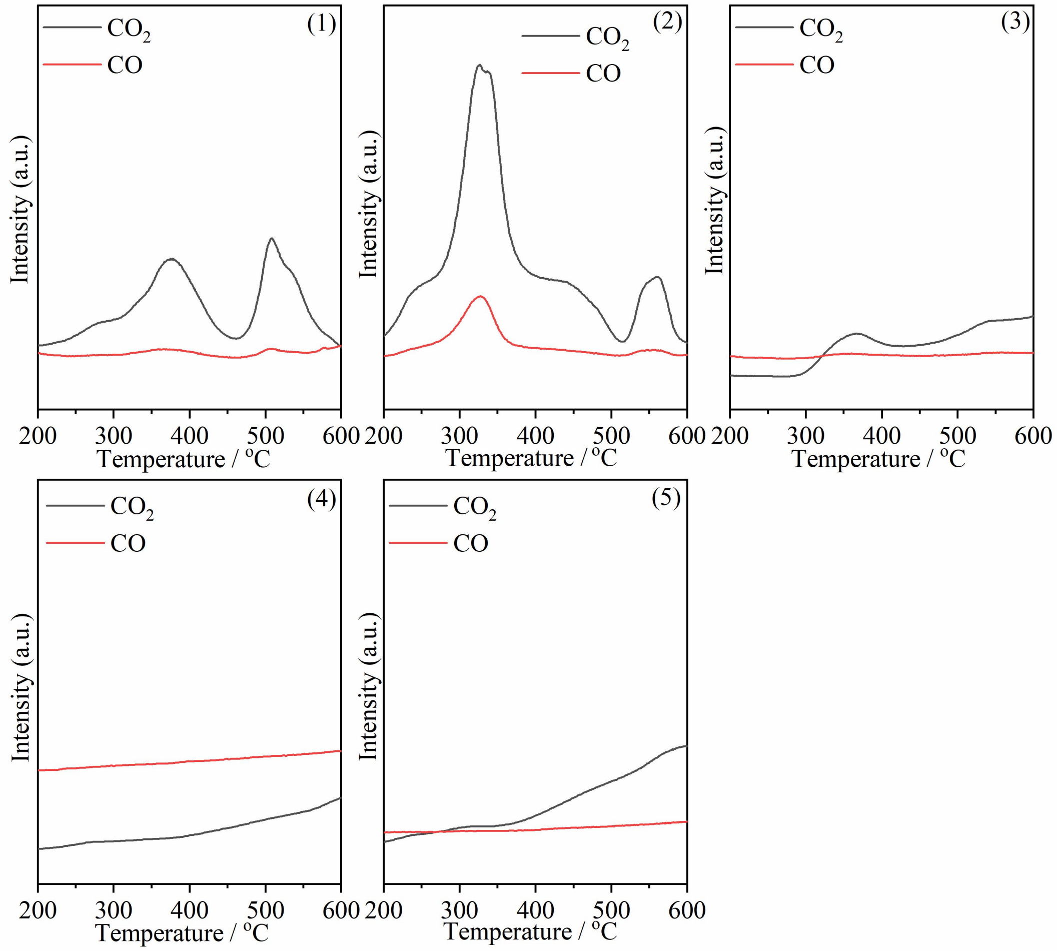

Figure S4. Comparison curve of CO and CO_2 production in Soot-TPR: (1) CM-Na; (2) CM-NaC; (3) CM-NC; (4) CM-N; (5) CM-3. (under the tight contact condition)

Characterization

The phase of the catalysts was detected on powder X-ray diffraction (XRD) on a Rigaku Ultima IV diffractometer (Rigaku, Japan) using Cu Kα radiation and operating at 40 kV and 40 mA. The detection range was 10-80 ° with a scanning step size of 0.02 °.

The textural properties of the cerium manganese catalysts were obtained by N_2 adsorption-desorption at -196 °C (liquid nitrogen temperature) on a Quantachrome evo sorption analyzer (Quantachrome, USA). The catalysts were vacuumized at 300 °C for 3 h before analysis. The specific surface area, total pore volume and pore diameter were calculated from Brunauer-Emmett-Teller (BET) and Barret-Joyner-Halenda (BJH) methods, respectively.

The morphological features of the cerium manganese catalysts were observed using a scanning electron microscope (SEM, Thermo Scientific Apreo 2C). And the elemental distribution of the catalysts was analyzed by the energy dispersive spectrometer (EDS, Oxford Ultim Max65). The samples were pretreated with gold spraying.

The Vis-Raman spectra of the catalysts were obtained on Thermo Scientific Dxr2xi Laser Raman spectrometer with an excitation wavelength of 532 nm. Data records range from 100 to 1000 cm^{-1}.

X-ray photoelectron spectra (XPS) were recorded on a Thermo Scientific^{TM} K-Alpha^{TM+} spectrometer equipped with Al Kα radiation as the excitation source. All peaks were corrected with C 1s peak binding energy at 284.8 eV.

Temperature-programmed reduction (H_2-TPR) measurements were investigated on XianQuan TP-5080-B. 100 mg of catalyst was firstly pretreated in the flow of Ar (30 mL/min) from room temperature to 450 °C (10 °C/min), maintained at 450 °C for 60 min. Followed by cooling down to 30 °C in the same atmosphere. Lastly, the catalyst was heated in 10% H_2/Ar from 30 °C to 900 °C with a heating rate of 10 °C/min.

O_2 temperature-programmed desorption (O_2-TPD) experiments were carried out on XianQuan TP-5080-B. Firstly, the catalysts were heated from RT to 400 °C in He atmosphere (30 mL/min) and kept at 400 °C for 1 h. Then followed by cooling down to 50 °C, after that the gas was switched to 3% O_2/He and held for 60 min. Afterwards it was turned to He atmosphere and kept for 60 min, and followed by heating to 800 °C at a rate of 10 °C/min.

Soot temperature programmed reduction (Soot-TPR) was carried out on autochem 2920-hiden HPR20 (Micrometetric Co.). Prior to the test, the catalyst and soot were carefully ground in a mortar for 5 min, which were then placed in a quartz reactor. The sample was treated in Ar with high purity at 150 °C for 60 min to remove any physically adsorbed impurities. Then, soot-TPR was carried out from 50 to 600 ℃ at a heating rate of 10 °C/min in Ar with high purity and flow rate of 30 mL/min . The formation of CO_x was detected by mass spectrum.

The in situ IR spectra were recorded on a Nicolet iS50 spectrometer equipped with an in-situ diffuse reflection cell. Firstly, the background spectrum without samples was collected in He at room temperature. Then, the mixture of soot and

catalyst (tight contact) was pressed into a wafer and placed in the in-situ infrared transport battery. The samples were treated at 200 °C in He (100 mL/min) for 60 min to remove weakly adsorbed species, and then cooled to room temperature. Finally, 5% O_2/He was introduced (100 mL/min) and the samples were heated to 500 °C at the rate of 10 °C/min. The temperature was maintained at 500 °C until the soot was burned out. The spectra were collected every 20 ℃.

Temperature programmed oxidation (O_2-TPO) experiments of catalyst and catalyst + soot were carried out under the same test conditions. These experiments were carried out on utochem 2920-hiden HPR20 (Micrometetric Co.). The sample was purged at a mixture of 5% O_2/He (30 mL/min) until the baseline was stable, and then it was heated from room temperature to 600 ℃ at a heating rate of 10 ℃ / min. Mass spectrum was used to detect the CO_x, CO and H_2O produced during the heating process
